# Associations of Plasma Concentrations of Dichlorodiphenyldichloroethylene and Polychlorinated Biphenyls with Prostate Cancer: A Case–Control Study in Guadeloupe (French West Indies)

**DOI:** 10.1289/ehp.1408407

**Published:** 2014-11-21

**Authors:** Elise Emeville, Arnaud Giusti, Xavier Coumoul, Jean-Pierre Thomé, Pascal Blanchet, Luc Multigner

**Affiliations:** 1 Institut National de la Santé et de la Recherche Médicale (INSERM), UMR 1085, IRSET, Pointe à Pitre, Guadeloupe, France; 2 Université de Rennes 1, Rennes, France; 3 Center for Analytical Research and Technology, Liege University, Liege, Belgium; 4 INSERM, UMR-S 747, Toxicologie Pharmacologie et Signalisation Cellulaire, Paris, France; 5 Université Paris Descartes, Sorbonne Paris Cité, Paris, France; 6 Service d’Urologie, Centre Hospitalier Universitaire Guadeloupe, Abymes, Guadeloupe, France

## Abstract

**Background:**

Long-term exposure to persistent pollutants with hormonal properties (endocrine-disrupting chemicals; EDCs) may contribute to the risk of prostate cancer (PCa). However, epidemiological evidence remains limited.

**Objectives:**

We investigated the relationship between PCa and plasma concentrations of universally widespread pollutants, in particular *p,p*´-dichlorodiphenyl dichloroethene (DDE) and the non-dioxin-like polychlorinated biphenyl congener 153 (PCB-153).

**Methods:**

We evaluated 576 men with newly diagnosed PCa (before treatment) and 655 controls in Guadeloupe (French West Indies). Exposure was analyzed according to case–control status. Associations were assessed by unconditional logistic regression analysis, controlling for confounding factors. Missing data were handled by multiple imputation.

**Results:**

We estimated a significant positive association between DDE and PCa [adjusted odds ratio (OR) = 1.53; 95% CI: 1.02, 2.30 for the highest vs. lowest quintile of exposure; *p*_trend_ = 0.01]. PCB-153 was inversely associated with PCa (OR = 0.30; 95% CI: 0.19, 0.47 for the highest vs. lowest quintile of exposure values; *p*_trend_ < 0.001). Also, PCB-153 was more strongly associated with low-grade than with high-grade PCa.

**Conclusions:**

Associations of PCa with DDE and PCB-153 were in opposite directions. This may reflect differences in the mechanisms of action of these EDCs; and although our findings need to be replicated in other populations, they are consistent with complex effects of EDCs on human health.

**Citation:**

Emeville E, Giusti A, Coumoul X, Thomé JP, Blanchet P, Multigner L. 2015. Associations of plasma concentrations of dichlorodiphenyldichloroethylene and polychlorinated biphenyls with prostate cancer: a case–control study in Guadeloupe (French West Indies). Environ Health Perspect 123:317–323; http://dx.doi.org/10.1289/ehp.1408407

## Introduction

Prostate cancer (PCa) is the second most common noncutaneous cancer among men worldwide and the leading noncutaneous cancer among men in developed countries (Center et al. 2012). Little is known about the risk factors associated with this cancer: Advancing age, ethnic origins, and a family history of PCa are the only established risk factors (Damber and Aus 2008; Hsing and Chokkalingam 2006). Many lifestyle-related risk factors, including westernization of eating habits and environmental chemical pollution, have been implicated, but their true roles in the etiology of PCa remain unclear (Damber and Aus 2008; Hsing and Chokkalingam 2006).

The effects of exposure to synthetic chemicals with hormonal properties in the environment, also called endocrine disruptors (EDCs), on prostate cancer development are also matters of debate (Diamanti-Kandarakis et al. 2009; Prins 2008; World Health Organization 2013). Models of PCa are not available for regulatory testing. This makes the identification of prostatic hormonal carcinogens very difficult, and forces researchers to rely on epidemiological studies. However, epidemiological evidence remains limited (Soto and Sonnenschein 2010).

Persistent organic pollutants, including *p,p*´-dichlorodiphenyldichloroethylene (DDE, the major and most stable metabolite of dichlorodiphenyltrichloroethane, DDT) and polychlorinated biphenyls (PCBs), have attracted attention because of their widespread presence both in the environment and in human beings, and their ability to interfere with hormone-regulated processes (Kelce et al. 1995; Plísková et al. 2005). Several epidemiological studies have investigated relationships between human exposure to DDE and PCBs, determined by blood measurement, and PCa, but most of them found no association (Aronson et al. 2010; Ritchie et al. 2003, 2005; Sawada et al. 2010). One study in the United States that included 65 PCa cases and 1,920 noncases reported a positive but not significant association with prevalent prostate cancer risk (Xu et al. 2010).

In a population-based case–control study of incident PCa patients and control subjects in the general population in Guadeloupe (French West Indies), environmental exposure to the estrogenic insecticide chlordecone was positively associated with PCa (Multigner et al. 2010). Here we report continuation of this study with a more detailed investigation of associations of DDE and PCBs with PCa.

## Methods

*Study population*. This study took place in Guadeloupe (French West Indies), a Caribbean archipelago, where most of the inhabitants are of African descent. The study included 709 consecutive incident cases of histologically confirmed PCa and 723 controls without PCa. Details of the selection of cases and controls have been described elsewhere (Multigner et al. 2010). Briefly, cases were recruited among subjects attending public and private urology clinics, with a recruitment area covering the entire territory of the Guadeloupe Archipelago. Controls were recruited from men participating in a free systematic health screening program open to the general population: Each year, a random population sample selected in accordance with the sex and age distribution of the general population was invited to participate in the program. Consecutive men ≥ 45 years of age were then invited to participate as controls in our case–control study of PCa, with selection according to the approximate age distribution of PCa diagnosis in Guadeloupe. Inclusion criteria for both cases and controls were current residence in Guadeloupe, both parents born on any Caribbean island with a population of predominantly African descent, and no hormone treatments or use of any other drugs known to influence the hypothalamic–pituitary–gonadal–adrenal axis (including inhibitors of 5α-reductase). Additional inclusion criteria for controls were normal findings upon digital rectal examination and total plasma PSA (prostate-specific antigen) concentration no higher than the 75th percentile for the corresponding age group of African-American men without clinical evidence of PCa (Morgan et al. 1996). Trained nurses obtained information for both patients and controls. Case patients were interviewed within 2 months of diagnosis, before receiving any kind of treatment. All subjects were interviewed in person to obtain information about their age (years), Caribbean origin (French West Indies, Haiti, or Dominica), education (primary, secondary, high school and higher), weight and height allowing the calculation of body mass index (BMI; kilograms per meter squared), waist and hip circumference allowing the calculation of waist-to-hip ratio (≤ 0.95, > 0.95), smoking (never, former, or current), alcohol consumption (never, former, or current), diabetes type 2 (no, yes), past residence in Western countries (no, yes), history of PSA screening (within the preceding 5 years: no, yes), and family history of PCa (first degree relatives: no, yes, not known). Participants were also asked to provide a blood sample between 0800 and 1000 hours, after overnight fasting. The study was approved by the Guadeloupean ethics committee for studies involving human subjects. Each participant provided written informed consent.

*Laboratory assays*. A high-resolution gas chromatograph (Thermo Quest Trace 2000, Milan, Italy) equipped with a Ni63 electron capture detection system was used to determine the serum concentrations of 24 PCB congeners (International Union of Pure and Applied Chemistry number): 6 dioxin-like (77, 105, 118, 126, 156, and 169) and 18 non-dioxin-like (18, 28, 52, 101, 110, 128, 138, 143, 149, 153, 170, 180, 183, 187, 194, 195, 206, and 209); *p,p*´-DDT, *p,p*´-DDD (dichlorodiphenyldichloroethane), and *p,p*´-DDE; the α, β, and γ isomers of hexachlorocyclohexane (HCH); and chlordecone. The limit of detection (LOD) was 0.05 μg/L for all organochlorine compounds except for chlordecone (0.06 μg/L). Detailed information about sampling, analysis, and quality assurance and control has been provided elsewhere (Debier et al. 2003; Multigner et al. 2010). Plasma total cholesterol and total triglyceride concentrations were determined enzymatically (DiaSys Diagnostic Systems GmbH, Holzheim, Germany), and total lipid concentration was calculated as previously described (Bernert et al. 2007).

*Statistical analysis*. We restricted our analysis to chemicals detected at a rate of more than 80% (DDE; PCB congeners 138, 153, and 180; and chlordecone) (Table 1). Correlations between concentrations of the frequently detected pollutants were explored by Spearman’s rank correlation analysis (see Supplemental Material, Table S1). The concentrations of the various PCBs were highly correlated (Spearman’s rho ≥ 0.76; all *p*-values < 0.001), so we restricted further analysis to PCB-153.

**Table 1:** Detection and concentrations of organochlorine pollutants in plasma samples from the study population [μg/L (μg/g lipids)].

Organochlorine^*a*^	Detection frequency (%)	Percentile				Maximum
		10th	25th	50th	75th	
Controls
*p,p*´-DDT	36.2	< LOD	< LOD	< LOD	0.07 (0.01)	1.7 (0.32)
*p,p*´-DDD	24.0	< LOD	< LOD	< LOD	0.04 (0.008)	0.84 (0.15)
*p,p*´-DDE	96.2	0.39 (0.07)	0.98 (0.18)	2.06 (0.38)	4.37 (0.75)	27.8 (6.7)
PCB-28	54.5	< LOD	< LOD	0.07 (0.01)	0.28 (0.05)	8.0 (1.4)
PCB-52	42.6	< LOD	< LOD	< LOD	0.28 (0.05)	12.7 (2.5)
PCB-101	52.1	< LOD	< LOD	0.05 (0.009)	0.13 (0.02)	1.1 (0.21)
PCB-118	59.2	< LOD	< LOD	0.08 (0.01)	0.20 (0.03)	3.3 (0.9)
PCB-138	97.4	0.18 (0.03)	0.31 (0.06)	0.53 (0.10)	0.90 (0.16)	12.2 (2.4)
PCB-153	98.2	0.24 (0.05)	0.48 (0.09)	0.85 (0.15)	1.47 (0.26)	16.5 (3.5)
PCB-180	97.4	0.23 (0.04)	0.39 (0.07)	0.64 (0.12)	1.03 (0.18)	10.3 (2.0)
α-HCH	35.9	< LOD	< LOD	< LOD	0.08 (0.01)	1.6 (0.32)
β-HCH	43.5	< LOD	< LOD	< LOD	0.09 (0.02)	1.9 (0.30)
γ-HCH	27.7	< LOD	< LOD	< LOD	0.08 (0.01)	1.8 (0.41)
Chlordecone	84.1	< LOD	0.17 (0.03)	0.42 (0.08)	0.83 (0.15)	49.2 (8.8)
Cases
*p,p*´-DDT	29.3	< LOD	< LOD	< LOD	0.06 (0.01)	2.5 (0.43)
*p,p*´-DDD	20.1	< LOD	< LOD	< LOD	0.03 (0.006)	0.99 (0.15)
*p,p*´-DDE	95.5	0.40 (0.08)	1.11 (0.22)	2.55 (0.50)	5.74 (1.07)	40.1 (6.6)
PCB-28	52.6	< LOD	< LOD	0.06 (0.01)	0.29 (0.05)	6.8 (1.1)
PCB-52	49.3	< LOD	< LOD	< LOD	0.38 (0.07)	6.7 (1.1)
PCB-101	51.2	< LOD	< LOD	0.05 (0.009)	0.13 (0.02)	1.2 (0.17)
PCB-118	62.0	< LOD	< LOD	0.08 (0.02)	0.18 (0.03)	2.4 (0.52)
PCB-138	97.9	0.17 (0.03)	0.30 (0.06)	0.54 (0.10)	0.87 (0.18)	6.7 (1.1)
PCB-153	98.8	0.23 (0.04)	0.41 (0.06)	0.78 (0.10)	1.24 (0.18)	8.4 (1.3)
PCB-180	97.2	0.25 (0.05)	0.37 (0.07)	0.62 (0.12)	0.90 (0.18)	6.2 (1.0)
α-HCH	28.5	< LOD	< LOD	< LOD	0.05 (0.01)	1.2 (0.20)
β-HCH	38.0	< LOD	< LOD	< LOD	0.11 (0.02)	2.2 (0.46)
γ-HCH	18.4	< LOD	< LOD	< LOD	< LOD	0.65 (0.13)
Chlordecone	82.8	< LOD	0.18 (0.03)	0.43 (0.08)	0.94 (0.18)	26.4 (4.1)
^***a***^PCB congeners 18, 77, 101, 105, 110, 126, 128, 143, 149, 156, 169, 170, 183, 187, 194, 195, 206, and 209 were below the LOD in all cases and controls.						

The odds ratio (OR) and 95% confidence intervals (CIs) for the association between PCa and organochlorines according to category of exposure were estimated using unconditional logistic regression. Organochlorines were categorized into quintiles according to the distribution in control subjects. Exposure levels equal to or below the LOD were included in the first (lowest) quintile.

Potential confounders were included as covariates in logistic models if they predicted case status (Table 2) and exposure (see Supplemental Material, Table S2) with *p* < 0.05. We also adjusted all models for total lipids (grams per liter), rather than modeling concentrations of the fat-soluble exposure of interest on a per-unit serum-lipid basis, because the latter approach may be prone to bias (Porta et al. 2009). For each exposure, we also considered the other contaminants as potential confounders. Spearman’s rank correlation coefficients between chlordecone and DDE concentrations and between chlordecone and PCB-153 concentrations were low (ρ = 0.05 and 0.07 in controls, and 0.04 and 0.07 in cases, respectively; see Supplemental Material, Table S1). Consequently, chlordecone was not considered as a confounder.

**Table 2:** Baseline characteristics of the study population.

Characteristic	Cases [no. (%)]	Controls [no. (%)]	*p*-Value^*a*^
Age (years) [mean (range)]	65.9 (52.6–79.1)	60.9 (48.0–77.1)	< 0.001
Caribbean origin
French West Indies	556 (96.5)	598 (91.3)	< 0.001
Haiti or Dominica	20 (3.5)	57 (8.7)
Education
Primary	349 (61.0)	362 (57.5)	0.04
Secondary	147 (25.7)	201 (32.0)	
High school and higher	76 (13.3)	66 (10.5)
Missing data	4	26
Body mass index (kg/m^2^)
< 25	241 (44.5)	306 (46.9)	0.54
25 to < 30	240 (44.3)	268 (41.1)
≥ 30	61 (11.2)	78 (12.0)
Missing data	34	3
Waist-to-hip ratio
≤ 0.95	196 (54.4)	455 (69.8)	< 0.001
> 0.95	164 (45.6)	197 (30.2)
Missing data	216	3
Smoking
Never	355 (62.2)	410 (62.9)	0.80
Former or current	216 (37.8)	242 (37.1)
Missing data	5	3
Alcohol consumption
Never	74 (13.0)	112 (17.4)	0.03
Former or current	494 (87.0)	536 (82.6)
Missing data	8	12
Type 2 diabetes
No	457 (81.5)	556 (87.4)	0.004
Yes	104 (18.5)	80 (12.6)
Missing data	15	19
Past residence in Western countries
No	403 (70.0)	498 (76.3)	0.01
Yes	173 (30.0)	155 (23.7)
Missing data	—	2
PSA screening history
No	278 (48.4)	572 (87.3)	< 0.001
Yes	296 (51.6)	83 (12.7)
Missing data	2	—
Family history of prostate cancer
No	317 (55.9)	498 (78.2)	< 0.001
Yes	144 (25.4)	66 (10.4)
Do not know	106 (18.6)	74 (11.4)
Missing data	9	18
Gleason score
< 7 or 3 + 4	462 (82.1)	—
> 7 or 4 + 3	101(17.9)	—
Missing data	9
Clinical stage (T, N, M)
T1c or T2 and N0 and M0	485 (87.4)	—
T3 or T4, or N+ or M+	70 (12.6)	—
Missing data	21
^***a***^*p*-Values were calculated using a two-sided chi-square test for a comparison of percentages or by a two-sided Student *t*-test for a comparison of means.			

Next, models of DDE as the primary exposure were adjusted for age (log linearity of age was not achieved, so age was categorized as quartiles according to the age distribution of the controls), waist-to-hip ratio, type 2 diabetes, alcohol consumption, total lipids, and PCB-153 (quintiles). Models of PCB-153 as the exposure were adjusted for the same covariates, plus Caribbean origin and past residence in a Western country, and DDE (quintiles). Sensitivity analyses were conducted including additional adjustment for BMI, PSA screening history, family history of PCa, and chlordecone. Additional sensitivity analyses were realized excluding any subject (*n* = 199), control or case, with a prediagnostic BMI < 18.5 or > 30.

Missing data for covariates varied from none to 2 (0.2%) for past residence in Western countries and for PSA screening history, 8 (0.6%) for smoking, 20 (1.6%) for alcohol, 27 (2.2%) for family history of PCa, 30 (2.4%) for education, 34 (2.8%) for diabetes, 37 (3.0%) for BMI, and 219 (17.8%) for waist-to-hip ratio. Missing data were handled by multiple imputations according to the methodology described by Rubin (1987) and Little and Rubin (1987) using chained equations (MICE; multiple imputation by chained equations) (Van Buuren et al. 1999; White et al. 2009). For the imputation procedure, we included the following characteristics: age, Caribbean origin, education, weight, height, waist and hip circumference, smoking, alcohol, diabetes, PSA screening history, family history of PCa, past residence in Western countries, total plasma lipids, all organochlorines, and case–control status. Five imputed data sets were generated using 20 cycles per imputation, and the main analyses were repeated using the imputed data. In addition, we performed sensitivity analyses substituting missing data with a missing value indicator variable, and by using complete case analyses restricted to participants with known values of all covariates. Tests for trends were performed by modeling categorical exposures as ordinal variables after assigning median values to each exposure category.

We considered possible interactions between organochlorine exposure and covariates in relation to PCa. The cross-product of covariates (BMI < 25 or > 25 kg/m^2^; waist-to-hip ratio ≤ 0.95 or > 0.95; smoking, never versus former or current; alcohol consumption, never versus former or current; diabetes type 2, yes, no; past residence in Western countries, yes, no; history of PSA screening, yes, no) and exposures (quintiles) was introduced in the logistic model. Subjects with missing values for the factors of interest were excluded from these analyses. We adjusted for the same covariates as the main model for each exposure. Consistent with the recommendations of Seaman et al. (2012), these analyses were restricted to participants with known values of all covariates. The *p*-value for interaction was calculated by the likelihood ratio test comparing the log-likelihood for the model with the interaction terms to the log-likelihood for the model without the interaction term. Interactions with a *p*-value for the cross-term product ≤ 0.20 were further assessed with stratified analyses.

Polytomous logistic regressions models were used to estimate associations between exposures and case subgroups (versus controls) according to grade (low grade: Gleason score < 7 or 3 + 4; high grade: Gleason score 4 + 3 or > 7) and clinical stage at diagnosis (tumor, nodes, metastases; localized stage: T1c or T2 and N0 and M0; advanced stage: T3 or T4, or N+ or M+). Exposures were categorized into tertiles according to the distribution in control subjects for theses analyses.

Using previously published data (Multigner et al. 2010), we reanalyzed the association between chlordecone exposure and PCa among participants included in the present analysis, with additional adjustment for plasma DDE and PCB-153. After analysis of quality control samples consisting of human plasma spiked with a series of concentrations of chlordecone, we defined the LOD for plasma chlordecone concentrations as 0.06 μg/L, rather than using an LOD of 0.25 μg/L, as in our previous analysis (Multigner et al. 2010).

SAS software version 9.3 (SAS Institute Inc., Cary, NC, USA) was used for analyses; all tests were two-sided, and *p*-values < 0.05 were considered statistically significant.

## Results

The results presented here were obtained from a study population comprising 576 of the 709 eligible PCa cases and 655 of the eligible 722 controls, from whom we were able to obtain blood samples and measure plasma organochlorine concentrations. The baseline characteristics of the study population are summarized in Table 2.

The adjusted OR was 1.53 (95% CI: 1.02, 2.30) for men in the highest quintile of DDE concentration compared with men in the lowest quintile (Table 3). The relationship between exposure and PCa was significant (*p*_trend_ = 0.01). This overall trend seems to be mainly driven by the OR for the highest versus lowest quintiles, because the other ORs were close to null. Results of sensitivity analyses were comparable with the primary analysis when missing data were modeled using missing value indicator categories; when we performed complete case analyses; and when BMI, PSA screening history, family history of PCa, or chlordecone exposure were included in the full model (see Supplemental Material, Table S3). Excluding subjects with BMI < 18.5 and > 30 resulted in a slight decrease in the OR (1.43; 95% CI: 0.93, 2.20), but the trend across exposure categories remained significant (*p*_trend_ = 0.04) (see Supplemental Material, Table S3).

**Table 3:** ORs (95% CIs) of prostate cancer according to quintile of DDE and PCB-153 exposure.

Exposure	Controls (*n*)	Cases (*n*)	Crude OR (95% CI)	Adjusted OR^*a*^ (95% CI)
DDE (μg/L)
< 0.79	131	106	1.0 (reference)	1.0 (reference)
0.79–1.62	130	96	0.91 (0.63, 1.62)	0.96 (0.66, 1.42)
1.63–2.89	133	111	1.03 (0.72, 1.48)	1.05 (0.71, 1.55)
2.90–5.18	131	104	0.98 (0.68, 1.41)	1.02 (0.67, 1.53)
≥ 5.19	130	159	1.51 (1.07, 2.13)	1.53 (1.02, 2.30)
*p*_Trend_			0.003	0.01
PCB-153 (μg/L)
< 0.41	132	141	1.0 (reference)	1.0 (reference)
0.41–0.69	132	109	0.77 (0.55, 1.09)	0.56 (0.38, 0.83)
0.70–1.07	134	135	0.94 (0.67, 1.32)	0.67 (0.46, 0.99)
1.08–1.70	131	110	0.79 (0.55, 1.11)	0.45 (0.30, 0.63)
≥ 1.71	126	81	0.60 (0.42, 0.87)	0.30 (0.19, 0.47)
*p*_Trend_			0.01	< 0.001
^***a***^For DDE: adjusted for age, waist-to-hip ratio, type 2 diabetes, alcohol, total plasma lipid concentration, and PCB-153. For PCB-153: adjusted for age, waist-to-hip ratio, Caribbean origin, past residence in Western countries, type 2 diabetes, total plasma lipid concentration, alcohol, and DDE. Missing values were imputed using a multiple imputation by chained equation (MICE) approach in five data sets.				

Contrary to what was observed for DDE, adjusted ORs relative to the lowest quintile of PCB-153 concentration all were significantly below 1 (OR = 0.30; 95% CI: 0.19, 0.47 for the highest versus lowest quintile) (Table 3). The overall trend for the association across exposure categories was significant (*p*_trend_ < 0.001). In sensitivity analyses, associations were comparable when missing data were modeled using missing value indicator categories; when restricted to a complete case analysis; and when additionally adjusted for BMI, PSA screening history, family history of PCa, or chlordecone exposure (see Supplemental Material, Table S4). Also, exclusion of subjects with BMI < 18.5 and > 30 did not greatly affect the ORs (see Supplemental Material, Table S4).

We did not find any evidence of effect modification (interaction *p*-values > 0.2, data not shown) except for family history of PCa and PCB-153 exposure (see Supplemental Material, Table S5). Associations between PCB-153 exposure and PCa were stronger in men without a family history of PCa, and the interaction terms, although not significant, were < 0.10 for the three highest quintiles of exposure.

Our next analyses considered clinical characteristics. The adjusted OR for cases with high-grade Gleason score was 1.92 (95% CI: 1.04, 3.54) for men in the highest tertile relative to men in the lowest tertile of DDE concentration (Table 4), but this was not significantly different from the corresponding OR value for cases with low-grade Gleason score (*p*_heterogeneity_ = 0.13). For PCB-153, a significant inverse association was observed among cases with low-grade Gleason score (OR = 0.35; 95% CI: 0.25, 0.51) for men in the highest tertile relative to men in the lowest tertile (Table 4); this was significantly different from what was observed for cases with high-grade score (*p*_heterogeneity_ = 0.04). No significant differences were observed between localized and advanced stage of PCa for either DDE or PCB-153 exposure.

**Table 4:** OR (95% CIs) for DDE and PCB-153, and prostate cancer by Gleason score and clinical stage.

Exposure	Controls (*n*)	Low grade (*n*)	Low-grade OR^*a*^ (95% CI)	High grade (*n*)	High-grade OR^*a*^ (95% CI)	*p*-Value^*b*^	Localized (*n*)	Localized OR^*a*^ (95% CI)	Advanced (*n*)	Advanced OR^*a*^ (95% CI)	*p*-Value^*c*^
DDE (μg/L)
< 1.37	218	144	1.0 (reference)	20	1.0 (reference)		145	1.0 (reference)	15	1.0 (reference)
1.37–3.41	218	151	1.06 (0.77, 1.47)	34	1.55 (0.85, 2.85)	0.23	160	1.11 (0.81, 1.52)	23	1.44 (0.69, 2.98)	0.50
≥ 3.42	219	167	1.18 (0.84, 1.65)	47	1.92 (1.04, 3.54)	0.13	180	1.26 (0.91, 1.76)	32	1.39 (0.66, 2.93)	0.83
*p*_Trend_			0.33		0.06			0.18		0.55
PCB-153 (μg/L)
< 0.60	218	183	1.0 (reference)	28	1.0 (reference)		181	1.0 (reference)	22	1.0 (reference)
0.61–1.24	216	174	0.78 (0.57, 1.06)	39	1.11 (0.63, 1.95)	0.22	189	0.83 (0.61, 1.14)	23	0.84 (0.42, 1.68)	0.97
≥ 1.25	221	105	0.35 (0.25, 0.51)	34	0.69 (0.37, 1.29)	0.04	115	0.38 (0.27, 0.55)	25	0.64 (0.30, 1.35)	0.19
*p*_Trend_			< 0.001		0.10			< 0.001		0.28	
^***a***^For DDE: adjusted for age, waist-to-hip ratio, alcohol, type 2 diabetes, total plasma lipid concentration, and PCB-153. For PCB-153: adjusted for age, waist-to-hip ratio, Caribbean origin, past residence in Western countries, type 2 diabetes, total plasma lipid concentration, alcohol, and DDE. Missing values were imputed using a multiple imputation by chained equation (MICE) approach in five data sets. ^***b***^*p-*Value from the Wald test for heterogeneity of respective β coefficients between low-grade and high-grade prostate cancer. ^***c***^*p-*Value from the Wald test for heterogeneity of respective β coefficients between localized and advanced-stage prostate cancer.											

Finally, we reanalyzed the association between chlordecone exposure and PCa: the OR was 1.65 (95% CI: 1.09, 2.48; *p*_trend_ = 0.01) for men in the highest quintile compared with men in the lowest quintile (see Supplemental Material, Table S6). Comparable results were observed if DDE or PCB-153 concentrations were included in the full model (OR = 1.64; 95% CI: 1.09, 2.47; *p*_trend_ = 0.01, and OR = 1.70; 95% CI: 1.12, 2.56; *p*_trend_ = 0.008, respectively) (see Supplemental Material, Table S6).

## Discussion

In our study population, the highest quintile of exposure to DDE, evaluated by determining plasma *p,p*´-DDE concentrations, was positively associated with incident PCa. By contrast, plasma PCB-153 was inversely associated with PCa, with significant negative associations for all quintiles above the reference level, and the strongest association with the highest quintile.

These results were obtained by studying a population with plasma concentrations consistent with the range of background environmental levels currently found in U.S. populations of similar age (Centers for Disease Control and Prevention 2009). The median value for plasma lipid–adjusted DDE (0.38 μg/g) and PCB-153 (0.15 μg/g) in our control population was, for DDE, in the same range as (0.27–0.94 μg/g) and, for PCB-153, slightly higher (0.04–0.09 μg/g) than those in control populations in other studies investigating the relationships between these pollutants, determined by blood measurement, and PCa (Aronson et al. 2010; Ritchie et al. 2003, 2005; Sawada et al. 2010; Xu et al. 2010). In the French West Indies, DDT has not been extensively used in agricultural supplies or for disease vector control. In addition, this territory has had only very limited industrial activities involving significant use or emission of PCBs. Consequently, exposure to these chemical pollutants is likely to be associated with background contamination of the food chain.

To our knowledge, this is the largest study to have investigated associations of DDE and PCBs with PCa based on biological measurements of exposure. Other strengths of this study include its population-based design, the consideration of co-exposure to other organochlorine compounds [particularly chlordecone, which has been found previously to be associated with the risk of PCa (Multigner et al. 2010)], case evaluation and exposure measurement within 2 months of diagnosis and before treatment, and using multiple imputation to handle missing data.

Our study also suffers some limitations inherent in the case–control design. Factors potentially generating bias must be considered, particularly those relating to differential errors in the measurement of disease or exposure. Case identification was based on unambiguous histological criteria, and controls were also selected on the basis of strict criteria, such as normal findings on digital rectal examination and PSA in the normal range for age, taking into account the ethnic background of the population.

The use of DDT and PCBs spread worldwide around the middle of the 20th century, so the study population has probably been exposed to these chemicals or their metabolites throughout much of their lifetimes. Single determinations of plasma organochlorine concentration provide an accurate reflection of the load of this compound in the body and are commonly used as an effective way to determine the extent of chronic exposure to these chemicals. However, questions have been raised about whether a single blood determination of persistent chemicals at the time of cancer diagnosis is a reliable indicator representing lifetime exposure, particularly for breast cancer (Verner et al. 2011). Nevertheless, unlike women, men are not subject to the mobilization of fat-soluble chemicals during pregnancy or breastfeeding that can significantly alter the pollutant load of the whole body. Any previous weight loss or gain, particularly if substantial, may modify the blood concentration of these pollutants. Unfortunately, we did not collect data for our study population about the gain or loss of body weight during adulthood. To overcome, albeit only in part, this lack of information, we performed a sensitivity analysis by excluding subjects who were underweight or obese: These individuals were, perhaps, the most likely to have changed weight significantly since the beginning of adulthood.

Few studies have investigated relationships between human exposure to DDE and PCBs, determined by blood measurement, and PCa, but all were inconclusive (Aronson et al. 2010; Ritchie et al. 2003, 2005; Sawada et al. 2010; Xu et al. 2010). Nevertheless, Xu et al. (2010) reported that ORs for the second and third tertiles of DDE exposure were 2.05 (95% CI: 0.76, 5.5) and 2.64 (95% CI: 0.92, 7.57), respectively. Nonsignificant inverse associations have been reported between PCBs and PCa in a Canadian case–control study (Aronson et al. 2010) and in a Japanese nested case–control study specifically addressing advanced-stage PCa (Sawada et al. 2010). An ecological study in Eastern Slovakia reported a lower incidence of PCa in a district with extensive environmental contamination from a former PCB production site, where residents presented higher concentrations of PCBs in blood levels than in a district without any history of PCB production and where residents had low blood concentrations of PCBs (Pavuk et al. 2004).

We investigated whether exposure to DDE or PCB-153 was associated with PCa aggressiveness. Gleason score and clinical stage at diagnosis are powerful predictors of the aggressiveness of PCa. In particular, patients with high-grade Gleason scores have lower metastasis-free survival and higher PCa-specific mortality. PCB-153 exposure appeared to be negatively associated with low-grade Gleason score. Screening procedures may have introduced distortions in the associations observed between exposures of interest and cancer outcomes if fewer cases had been included in the absence of screening (Weiss 2003). In our study population, the prevalence of PSA screening among PCa cases with low-grade Gleason score was 76.7% but among PCa cases with a high-grade score, it was only 10%. Also, we found that additional adjustment for PSA screening did not change the risk estimates (data not shown). These various observations suggest that PCB-153 exposure may truly decrease the occurrence of low-grade PCa without changing the occurrence of high-grade forms. Koutros et al. (2013) have suggested that the different associations between chemical exposures (i.e., pesticides) and PCa aggressiveness may be consequences of different roles of such exposures in the prostatic carcinogenesis (for example, earlier initiation stage vs. prostate cancer progression). However, it has not been established that nonaggressive and aggressive forms of PCa are etiologically and pathogenically similar.

Finally, we found that the negative association between PCB-153 and PCa was stronger among subjects without a family history of PCa than among those with such a family history. Because the interaction terms were not strictly significant and number of cases with a family history of PCa was very small, these results should be interpreted with caution. This result differs from those reported for various other organochlorine or pesticide exposures: Increased risks have been observed among subjects with a family history of PCa, possibly due to genetic susceptibility (Alavanja et al. 2003; Christensen et al. 2010; Lynch et al. 2009; Mahajan et al. 2006; Multigner et al. 2010). Overall, exposure to PCB-153 appears to be inversely associated with less aggressive prostate cancer and tends to be most strongly associated among subjects without a family history of PCa; such patients have a better prognosis than those with a family history (Kupelian et al. 2006).

Mainly on the basis of data from animal experiments, the International Agency for Research on Cancer (IARC) currently classifies DDT as “possibly carcinogenic to humans” and PCBs (because of their positive association with melanoma in humans) as “probably carcinogenic to humans” (IARC 1991). Both are classified as “reasonably anticipated to be human carcinogens” by the National Toxicology Program (2014). Thus, the observation from this study that PCa is positively associated with DDE and negatively associated with PCB-153 is unexpected; however, these findings may reflect differences in the hormonal properties of DDE and PCB-153 and their effects on prostate development, as discussed below.

DDE displays anti-androgenic effects *in vivo*, as assessed from changes in the weights of androgen-responsive tissues (Owens et al. 2007). These effects are probably mediated by competitive binding to the androgen receptor (AR) and/or inhibition of AR-dependent gene expression (Kelce et al. 1995, 1997). In adult healthy subjects without PCa, DDE exposure is negatively associated with serum concentration of dihydrotestosterone (Emeville et al. 2013), suggesting that DDE could also indirectly affect androgen signaling. However, DDE, like many other EDCs, has mixed actions on different members of the steroid receptor superfamily. DDE also exerts agonistic activity on estrogen receptor alpha (ERα) (Li et al. 2008). ERα mediates adverse effects of estrogen on the prostate, including aberrant proliferation, inflammation, and malignancy (Ellem and Risbridger 2009). It is therefore difficult to predict the net effect of DDE on the prostate given potential effects on both AR and ERα (Carruba 2007; Ellem and Risbridger 2010).

Unlike dioxin-like PCBs, non-dioxin-like PCBs, which are the most common prevalent PCBs in the environment (McFarland and Clarke 1989), do not interact substantially with the aryl hydrocarbon receptor and may act through different pathways, such as steroid hormone signaling (Cooke et al. 2001). Experimental studies using various animal models have shown that PCB-153—the PCB congener most commonly found in animal and human tissues, due to its high persistence and low environmental degradability (Safe 1993)—has pro-estrogenic activities (Cooke et al. 2001; Dickerson et al. 2011; Hansen 1998). However, PCBs have also been reported to be anti-estrogenic in both reporter gene and MCF-7 cell proliferation assays (Plísková et al. 2005) and to decrease ER-mediated activity in ER-CALUX bioassays (Oh et al. 2007). Thus, the actions of non-dioxin-like PCBs on ER pathways are complex and depend on the ER subtypes that are being activated or antagonized. Moreover, the non-genomic ER pathways should also be considered. In MCF-7 cells, PCB-153 induces the mitogen-activated protein kinase involved in the extracellular signal-regulated kinase (ERK) 1/2 signaling pathways (Radice et al. 2008). Several isothiocyanates from cruciferous vegetables and polyphenols from green or black tea inhibit human PCa cell proliferation (Gupta et al. 2001; Melchini et al. 2013). Interestingly, the antiproliferative effects of these substances seem to be mediated by ERK 1/2 phosphorylation (Melchini et al. 2013; Siddiqui et al. 2004).

In summary, the modes of action of DDE and non-dioxin-like PCBs need to be investigated, particularly as involves all the various steroid receptor pathways to improve our understanding of their involvement in the proliferation or inhibition of PCa cells.

More than 20 years after the endocrine disruption concept first emerged (Colborn et al. 1993), this issue is still the subject of debate (Bergman et al. 2013; Dietrich et al. 2013; Gore et al. 2013). For instance, it has been reported that some EDCs have unexpected and potent effects at very low doses and/or do not generate the standard monotonic dose response curves seen for other types of compounds (Fagin 2012). Whether the interplay between different receptor mechanisms can generate unusual dose–response relationships and/or explains the associations we estimated for PCB-153 remains to be elucidated.

Caution is required in the interpretation of our findings. The possibility that our findings were confounded by unmeasured exposures or could be explained by reverse causality cannot be excluded. However, the possible influence, if any, of PCa on organochlorine concentrations in blood remains to be studied, and nothing is known about any underlying mechanism. Also, we cannot exclude the possibility that our findings, particularly for PCBs, may have resulted from selection bias associated with uncontrolled or unmeasured common causes of competing outcomes of PCB-related diseases and PCa (Thompson et al. 2013).

## Conclusions

In our study population of men of African descent from the French West Indies, DDE exposure was positively associated with PCa, whereas PCB-153 exposure was negatively associated with PCa. PCB-153 exposure was also inversely associated with less aggressive forms of the disease. These contrasting associations may be related to the different and sometimes multiple modes of hormonal action attributed to these two classes of pollutants. Our findings add complexity to the already controversial issue of EDCs and their suspected effects on human health. Replication of these observations in other populations, as well as mechanistic studies, is needed before any causal link can be established.

## Supplemental Material

(270 KB) PDF
