# Supplementary material for: Associations of Plasma Concentrations of Dichlorodiphenyldichloroethylene and Polychlorinated Biphenyls with Prostate Cancer: A Case–Control Study in Guadeloupe (French West Indies)
Source: Environ Health Perspect. 2014 Nov 21;123(4):317–23. doi: 10.1289/ehp.1408407 (PMC4384204; doi:10.1289/ehp.1408407)
Supplement: (270 KB) PDF [file ehp.1408407.s001.508.pdf]

Environmental Health Perspectives doi:10.1289/ehp.1408407

Supplemental Material

Associations of Plasma Concentrations of Dichlorodiphenyldichloroethylene and Polychlorinated Biphenyls with Prostate Cancer: A Case–Control Study in Guadeloupe (French West Indies)

Elise Emeville, Arnaud Giusti, Xavier Coumoul, Jean-Pierre Thomé, Pascal Blanchet, and Luc Multigner

Table S1. Spearman's rank correlation analysis of the relationship between concentrations of frequently detected (Limit of detection >80 %) pollutants in plasma samples from controls subjects and cases patients.

**Controls subjects (n = 655)**	**PCB138**	**PCB153**	**PCB180**	**Chlordecone**
DDE	*r* = 0.48<br>P <0.001	*r* = 0.38<br>P <0.001	*r* = 0.26<br>P <0.001	*r* = 0.05<br>P = 0.18
PCB138	-	*r* = 0.88<br>P < 0.001	*r* = 0.84<br>P <0.001	*r* = 0.10<br>P = 0.007
PCB153	-	-	*r* = 0.81<br>P <0.001	*r* = 0.07<br>P = 0.06
PCB180	-	-	-	*r* = 0.09<br>P = 0.002
**Cases patients (n = 576)**				
DDE	*r* = 0.50<br>P <0.001	*r* = 0.44<br>P <0.001	*r* = 0.33<br>P < 0.001	*r* = 0.04<br>P = 0.29
PCB138	-	*r* = 0.84<br>P <0.001	*r* = 0.82<br>P <0.001	*r* = 0.12<br>P = 0.003
PCB153	-	-	*r* = 0.76<br>P <0.001	*r* = 0.07<br>P = 0.09
PCB180	-	-	-	*r* = 0.14<br>P = 0.0008

Table S2. Geometric means of DDE and PCB153 plasma concentrations according to study population characteristics.

Characteristic	DDE Geometric means (CI 95%)	P value[a]	PCB153 Geometric means (CI 95%)	P-value[a]
**Caribbean origin**		0.42		<0.001
French West Indies	1.96 (1.78, 2.15)		0.78 (0.73, 0.83)	
Haiti or Dominica	1.71 (1.19, 2.45)		0.28 (0.22, 0.35)	
**Education**		0.59		0.61
Primary	1.84 (1.62, 2.09)		0.78 (0.64, 0.95)	
Secondary	2.00 (1.67, 2.40)		0.72 (0.63, 0.81)	
High school and higher	2.02 (1.62, 2.68)		0.72 (0.66, 0.78)	
**Body mass index** (kg/m²)		<0.001		0.03
<25	1.59 (1.38, 1.84)		0.68 (0.62, 0.75)	
25 - <30	2.06 (1.78, 2.40)		0.79 (0.72, 0.88)	
≥30	3.02 (2.27, 4.02)		0.73 (0.60, 0.89)	
**Waist-to-hip-ratio**		<0.001		0.01
≤0.95	1.66 (1.47, 1.88)		0.70 (0.64, 0.77)	
>0.95	2.50 (2.12, 2.95)		0.83 (0.74, 0.93)	
**Smoking**		0.35		0.01
Never	1.89 (1.68, 2.12)		0.69 (0.64,0.75)	
Former or current	2.04 (1.76, 2.36)		0.80 (0.73,0.89)	
**Alcohol consumption**		0.01		0.009
Never	1.53 (1.21, 1.92)		0.62 (0.52, 0.72)	
Former or current	2.02 (1.83, 2.23)		0.75 (0.70, 0.81)	
**Type 2 diabetes**		<0.001		0.02
No	1.81 (1.64, 1.99)		0.72 (0.67, 0.77)	
Yes	2.71 (2.15, 3.42)		0.86 (0.73, 1.01)	
**PSA screening history**		0.30		0.44
No	1.83 (1.69, 2.10)		0.72 (0.67, 0.78)	
Yes	2.06 (1.75, 2.43)		0.76 (0.68, 0.85)	
**Family history of prostate cancer**		0.66		0.36
No	1.95 (1.73, 2.20)		0.75 (0.69, 0.81)	
Yes	1.94 (1.53, 2.46)		0.67 (0.57, 0.79)	
Do not know	1.75 (1.36, 2.26)		0.73 (0.61, 0.87)	
**Past residence in Western countries**		0.71		<0.001
No	1.92 (1.67, 2.37)		0.65 (0.61, 0.70)	
Yes	1.99 (1.73, 2.14)		1.00 (0.89, 1.13)	

[a]P-values were calculated using ANOVA tests (two-sided).

Table S3. Sensitivity analysis of the association between DDE exposure and prostate cancer.

	DDE exposure <0.79 µg/L	DDE exposure 0.79-1.62 µg/L	DDE exposure 1.63-2.89 µg/L	DDE exposure 2.90-5.18 µg/L	DDE exposure ≥5.19 µg/L	P-Trend
**Controls subjects/cases patients (n)**	131/106	130/96	133/111	131/104	130/159	
**Adjusted[a]** OR (95% CI)	1.0	0.96 (0.66, 1.42)	1.05 (0.71, 1.55)	1.02 (0.67, 1.53)	1.53 (1.02, 2.30)	0.01
**Adjusted[b]** OR (95% CI)	1.0	1.00 (0.63, 1.56)	1.05 (0.66, 1.64)	1.01 (0.63, 1.62)	1.64 (1.04, 2.59)	0.007
**Adjusted[c]** OR (95% CI)	1.0	1.04 (0.65, 1.63)	1.03 (0.64, 1.64)	0.99 (0.61, 1.61)	1.73 (1.08, 2.78)	0.006
**Adjusted[a] including BMI** OR (95% CI)	1.0	0.96 (0.65, 1.43)	1.05 (0.70, 1.55)	1.01 (0.67, 1.53)	1.54 (1.03, 2.31)	0.01
**Adjusted[a] including family history of prostate cancer** OR (95% CI)	1.0	1.00 (0.66, 1.51)	1.08 (0.72, 1.62)	1.01 (0.66, 1.55)	1.64 (1.08, 2.48)	0.006
**Adjusted[a] including PSA screening history** OR (95% CI)	1.0	1.00 (0.70, 1.64)	1.06 (0.69, 1.64)	1.05 (0.67, 1.65)	1.55 (1.00, 2.41)	0.03
**Adjusted[a] including chlordecone** OR (95% CI)	1.0	0.92 (0.62, 1.37)	1.04 (0.70, 1.54)	1.03 (0.68, 1.56)	1.51 (1.01, 2.27)	0.01
**Adjusted[a,d] excluding subjects with BMI <18.5 and subjects with BMI >30** OR (95% CI)	1.0	1.00 (0.66, 1.54)	0.99 (0.65, 1.53)	1.07 (0.70, 1.66)	1.43 (0.93, 2.20)	0.04

[a]Adjusted for age, waist-to-hip-ratio, type 2 diabetes, alcohol, total plasma lipid concentration and PCB153, with missing values imputed using a Multiple Imputation by Chained Equation (MICE) approach in five data sets. [b]Adjusted for age, waist-to-hip-ratio, type 2 diabetes, alcohol, total plasma lipid concentration and PCB153, with missing value indicator categories. [c]Adjusted for age, waist-to-hip-ratio, type 2 diabetes, alcohol, total plasma lipid concentration and PCB153, and restricted to controls and cases with complete datasets. No. of controls/cases were, 125/64, 126/62, 126/63, 125/59, and 120/101 for quintiles 1 to 5 respectively. [d]No. of controls/cases were, 121/95, 108/81, 124/ 87, 110/88, and 110/120 for quintiles 1 to 5 respectively.

Table S4. Sensitivity analysis of the association between PCB153 exposure and prostate cancer.

	PCB153 exposure <0.41 µg/L	PCB153 exposure 0.41-0.69 µg/L	PCB153 exposure 0.70-1.07 µg/L	PCB153 exposure 1.08-1.70 µg/L	PCB153 exposure ≥1.71 µg/L	P-Trend
**Controls subjects/cases patients (n)**	132/141	132/109	134/135	131/110	130/159	
**Adjusted**[a] OR (95% CI)	1.0	0.56 (0.38, 0.83)	0.67 (0.46, 0.99)	0.45 (0.30, 0.63)	0.30 (0.19, 0.47)	<0.001
**Adjusted** [b] OR (95% CI)	1.0	0.65 (0.42, 1.01)	0.68 (0.43, 1.05)	0.43 (0.27, 0.70)	0.31 (0.18, 0.52)	<0.001
**Adjusted**[c] OR (95% CI)	1.0	0.64 (0.44, 1.09)	0.74 (0.47, 1.65)	0.40 (0.25, 0.66)	0.29 (0.17, 0.50)	<0.001
**Adjusted**[a] **including BMI** OR (95% CI)	1.0	0.56 (0.38, 0.83)	0.67 (0.46, 0.99)	0.45 (0.30, 0.69)	0.30 (0.19, 0.47)	<0.001
**Adjusted**[a] **including family history of prostate cancer** OR (95% CI)	1.0	0.60 (0.40, 0.89)	0.67 (0.45, 1.00)	0.48 (0.31, 0.73)	0.31 (0.19, 0.49)	<0.001
**Adjusted**[a] **including PSA screening history** OR (95% CI)	1.0	0.60 (0.40, 0.92)	0.68 (0.45, 1.03)	0.42 (0.26, 0.65)	0.31 (0.19, 0.51)	<0.001
**Adjusted**[a] **including chlordecone** OR (95% CI)	1.0	0.54 (0.37, 0.80)	0.64 (0.43, 0.65)	0.43 (0.28, 0.65)	0.28 (0.18, 0.45)	<0.001
**Adjusted**[a,d] **excluding subjects with BMI <18.5 and subjects with BMI >30** OR (95% CI)	1.0	0.65 (0.43, 1.00)	0.73 (0.47, 1.11)	0.54 (0.36, 0.86)	0.31 (0.18, 0.51)	<0.001

[a]Adjusted for age, waist-to-hip-ratio, diabetes type 2, Caribbean origin, past residence in western countries, total plasma lipid concentration and DDE, with missing values imputed using a Multiple Imputation by Chained Equation (MICE) approach in five data sets. [b]Adjusted for age, waist-to-hip-ratio, diabetes type 2, Caribbean origin, past residence in western countries, total plasma lipid concentration and DDE, with missing value indicator categories. [c]Adjusted for age, waist-to-hip-ratio, diabetes type 2, Caribbean origin, past residence in western countries, total plasma lipid concentration and DDE, and restricted to controls and cases with complete datasets for. No. of controls/cases were 119/129, 111 /94, 123/118, 111/ 101, and 113/73 for quintiles 1 to 5 respectively. [d]No. of controls/cases were, 115/110, 109/90, 117/108, 108/97, and 112/66 for quintiles 1 to 5 respectively.

Table S5. PCB153 exposure and prostate cancer according to family history of prostate cancer.

PCB153 exposure (µg/L)	No family history No. controls	No family history No. cases	No family history: Adjusted OR[a] (95% CI)	With family history No. controls	With family history No. cases	With family history: Adjusted OR [a] (95% CI)	P-Interaction
<0.41	87	47	1.0	17	22	1.0	
0.41-0.69	99	38	0.42 (0.23, 0.78)	12	14	0.98 (0.29, 3.32)	0.31
0.70-1.07	97	41	0.47 (0.23, 0.80)	14	23	1.91 (0.60, 6.10)	0.07
1.08-1.70	99	36	0.30 (0.16, 0.59)	7	15	1.28 (0.33, 5.03)	0.10
≥1.71	96	28	0.20 (0.10, 0.41)	10	14	0.84 (0.20, 3.40)	0.09
**P-Trend**			<0.001			0.88	

[a]Adjusted for age, waist-to-hip-ratio, Caribbean origin, past residence in western countries, type 2 diabetes, total plasma lipid concentration, alcohol and DDE, and restricted to controls and cases with complete datasets for.

Table S6. ORs (95% CIs) of prostate cancer according to quintile of chlordecone.

	**Chlordecone exposure <0.13 µg/L**	**Chlordecone exposure 0.13-0.30 µg/L**	**Chlordecone exposure 0.31-0.51 µg/L**	**Chlordecone exposure 0.52-1.02 µg/L**	**Chlordecone exposure ≥1.03 µg/L**	**P-Trend**
**Controls subjects/cases patients (n)**	132/113	128/85	131/127	134/121	130/130	
**Adjusted**[a] OR (95% CI)	1.0	1.00 (0.65, 1.54)	1.47 (0.98, 2.21)	1.41 (0.94, 2.13)	1.65 (1.09, 2.48)	0.01
**Adjusted**[a] **including DDE** OR (95% CI)	1.0	1.01 (0.66, 1.56)	1.48 (0.99, 2.22)	1.41 (0.93, 2.12)	1.64 (1.09, 2.47)	0.01
**Adjusted**[a] **including PCB153** OR (95% CI)	1.0	0.98 (0.64, 1.52)	1.51 (1.01, 2.27)	1.45 (0.96, 2.27)	1.70 (1.12, 2.56)	0.008

[a]Adjusted for age, waist-to-hip-ratio, PSA screening history, and total plasma lipid concentration. Missing values were imputed using a Multiple Imputation by Chained Equation (MICE) approach in five datasets.
